# The first complete mitochondrial genome of *Strigea falconis* (Digenea: Strigeidae) reveals six tandemly repeated *trnE*-containing units and provides mt evidence for the non-monophyly of the family Strigeidae

**DOI:** 10.1186/s12864-026-13156-1

**Published:** 2026-07-11

**Authors:** Ya Zhang, Ya Tu, Chaoqun Yao, Yi-Liu Liu, Yuan-Ping Deng, Ying Xun, Rong Li, Guo-Hua Liu, Yi-Tian Fu

**Affiliations:** 1 Research Center for Parasites & Vectors, College of Veterinary Medicine, Hunan Agricultural University, Changsha, Hunan 410128 China; 2 Chinese Medicinal Materials Breeding Innovation Center, Yuelushan Laboratory, Changsha, Hunan 410128 China; 3 Beijing Wildlife Rescue and Rehabilitation Center, Beijing, 101300 China; 4 One Health Center for Zoonoses and Tropical Infectious Diseases, Ross University School of Veterinary Medicine, Basseterre, Saint Kitts And Nevis; 5 College of Animal Science and Technology, Tarim University, Alar, Xinjiang 843300 China

**Keywords:** *Strigea falconis*, Mitochondrial genome, Tandem repeats, Phylogenetic analysis, Diplostomida

## Abstract

**Background:**

Phylogenetic relationships among members in the order Diplostomida remain contentious, with mitochondrial (mt) and nuclear genomic data often yielding conflicting topologies. A major limitation is the availability of only a few mt genomes from the type genus *Strigea*, hindering a robust test of the monophyly of the family Strigeidae and the order Diplostomida.

**Results:**

The mt genome of *S. falconis* was completely sequenced for the first time, which was a circular molecule of 16,872 bp in length, encoding the typical set of 36 mt genes and six duplicate tRNA-Glu genes. Notably, there were seven identical and consecutive tandem repeat units each consist of a 169 bp non-coding region followed by a *trnE* gene in the newly assembled genome. Phylogenomic analyses based on concatenated predicted amino acid sequences of 12 proteins robustly placed *S. falconis* in the same clade as *Apharyngostrigea pipientis*. Crucially, the family Strigeidae was not recovered as monophyletic. Instead, two species within Strigeidae, *Cardiocephaloides medioconiger* and *Cotylurus marcogliesei*, clustered with representatives of Diplostomidae, providing mt evidence for the paraphyly of Strigeidae under the current sampling.

**Conclusions:**

The newly sequenced mt genome of *S. falconis* reveals a previously unreported six-copy tandem repeat of *trnE*-containing units among currently available diplostomoid mt genomes. Phylogenetic analyses based on mt protein-coding genes provide additional mt evidence that the family Strigeidae was not recovered as monophyletic under the present taxon sampling. However, because mt genomes represent a single maternally inherited linkage group, broader taxon sampling, independent nuclear phylogenomic data, and explicit sensitivity analyses will be required to confirm these relationships and guide any formal systematic revision.

**Supplementary Information:**

The online version contains supplementary material available at https://doi.org/10.1186/s12864-026-13156-1.

## Introduction

The Digenea (Platyhelminthes: Trematoda) subclass contains over 18,000 known species of flukes that infect all major vertebrates. They all exhibit a complex life cycle involving at least two hosts [1]. Digenean phylogeny, which is pivotal in understanding their evolution, ecology, and epidemiology, has been a subject of long-standing debate and continuous revision. A landmark work based on morphology and nuclear ribosomal RNA (rDNA) genes by Olson and colleagues [2] proposed an influential classification system for Digenea, in which the order Diplostomida was erected to include Schistosomatoidea, Brachylaimoidea, and Diplostomoidea.

However, accumulating molecular evidence, particularly from complete mitochondrial (mt) genomes, has increasingly questioned aspects of this classification. Mounting phylogenomic evidence reveals a profound mito-nuclear discordance, wherein mt genome analyses robustly suggest the paraphyly of Diplostomida [3, 4]. Specifically, the Schistosomatoidea establishes as an early-diverging lineage, whereas the Brachylaimoidea and Diplostomoidea form a clade sister to the order Plagiorchiida [5, 6]. This incongruence points to an ancient rapid radiation event or differential selection pressures, presenting a central unresolved problem in trematode evolutionary history [7, 8].

This phylogenetic incongruence extends to the superfamily level. Within the Diplostomoidea, the monophyly of its two major families, Strigeidae and Diplostomidae, remains contentious. Studies incorporating limited mt genomic data have suggested that these two families are not monophyletic, with some strigeid genera such as *Cardiocephaloides*, *Cotylurus*, and *Apharyngostrigea* robustly nesting within or as sister to diplostomid clades, thereby rendering one or both families paraphyletic [7, 9, 10]. A critical and persistent limitation underpinning this uncertainty is lack of mt genome from *Strigea*, the type genus of the Strigeidae family. The position of this phylogenomic anchor is indispensable for any definitive test of Strigeidae monophyly and for resolving the true boundaries between these families, a principle widely recognized in phylogenomic studies [4, 11].

*Strigea falconis* Szidat, 1928, a common intestinal parasite of raptors and other birds with a Holarctic distribution, represents an ideal candidate to fill this pivotal data gap [12–14]. Beyond its phylogenetic significance, it is a pathogen of veterinary importance as their infections can result in morbidity and mortality in captive or immunocompromised avian hosts [15]. The aims of the current study include: (i) to sequence the complete mt genome of *Strigea* spp.; (ii) to clarify the mt phylogenetic placement for the type genus *Strigea*; (iii) to assess the monophyly of Strigeidae based on available mt genome data; and (iv) to re-evaluate the deep evolutionary relationships within the Diplostomida to address the mito-nuclear discordance at the heart of digenean systematics.

## Results

### Mt *cox1* gene sequence

The sizes of PCR products of the whole ten samples matched the expected sizes (~ 450 bp). Intraspecific sequences analysis showed that the similarities of the mt *cox1* gene sequences obtained in the present study were 100%, all of which were 100% identical to that of *S. falconis* from *Buteo buteo* (Common buzzard) originated from the Czech Republic (GenBank accession No. MF628045), confirming morphological identification.

### Mt genome organization, content and nucleotide composition

**Fig. 1:**
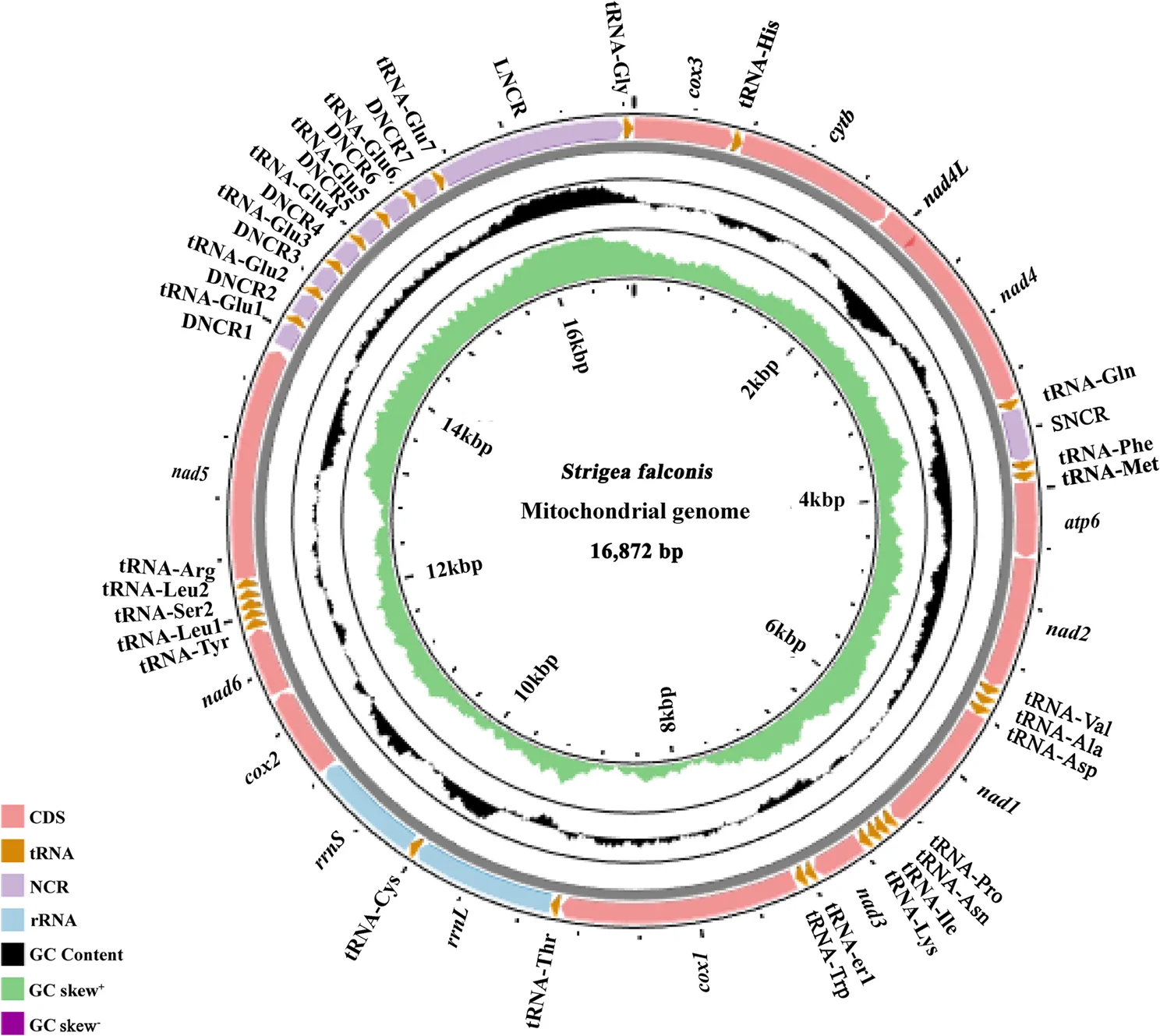
Organization of the mitochondrial genome of *Strigea falconis*. The scale is approximate. All genes have standard nomenclature except for the 24 tRNA genes, which are designated by the one-letter code for the corresponding amino acid, with numerals differentiating each of the two leucine- and serine-specifying tRNAs (L_1_ and L_2_ for codon families CTN and TTR, respectively; S_1_ and S_2_ for codon families TCN and AGN, respectively). All genes are transcribed in the clockwise direction. ‘NCR’ indicates the non-coding region

The complete mt genome of *S. falconis* is a circle of 16,872 bp (GenBank Acc. No. PP112874) (Fig. 1). It contains 42 genes in total, including 12 protein-coding genes (PCGs) (*atp8* is absent), 22 typical tRNA genes and six additional duplicated *trnE* genes, and two rRNA genes (*rrnL* and *rrnS*). The structure of this tandem repeat region was further verified by long-read sequencing of the fifth long-range PCR amplicon (Fig. S1). All genes are encoded on the same strand, a commonly shared feature in trematodes (Table 1). The gene order is identical to that reported for *C. marcogliesei* and *C. medioconiger* [7]. The overall nucleotide composition is A (17.3%), T (47.8%), G (25.8%), and C (9.1%), resulting in a high A + T content of 65.0%. This bias is reflected across genomic features (Table 2). The mt genome exhibits a strong negative AT-skew (-0.469) and a strong positive GC-skew (0.478), indicating a strand-specific bias in nucleotide content. Long-range PCR results using five pairs of specific primers were shown in Figure S1.

**Table 1:** The organization of the mitochondrial genome of *Strigea falconis*

Gene	Positions	Size (bp)	Number of aa^a^	Ini/Ter codons^b^	Anticodons	In^c^
*cox3*	1-685	685	228	ATG/T		0
tRNA-His (H)	686–752	67			GTG	+ 3
*cytb*	756–1857	1102	367	GTT/T		+ 6
*nad4L*	1864–2127	264	87	GTG/TAA		-40
*nad4*	2088–3386	1299	432	ATG/TAG		+ 3
tRNA-Gln (Q)	3390–3453	64			TTG	0
SNCR	3454–3812	359				0
tRNA-Phe (F)	3813–3878	68			GAA	+ 6
tRNA-Met (M)	3885–3952	68			CAT	+ 3
*atp6*	3956–4471	516	171	GTG/TAG		+ 5
*nad2*	4477–5380	904	301	ATG/T		0
tRNA-Val (V)	5381–5447	67			TAC	+ 11
tRNA-Ala (A)	5459–5520	62			TGC	+ 11
tRNA-Asp (D)	5532–5599	68			GTC	+ 3
*nad1*	5603–6508	906	301	GTG/TAG		+ 6
tRNA-Pro (P*)*	6515–6577	63			TGG	+ 8
tRNA-Asn (N)	6586–6651	66			GTT	0
tRNA-Ile (I)	6652–6718	67			GAT	+ 9
tRNA-Lys (K)	6728–6795	68			CTT	+ 4
*nad3*	6800–7156	357	118	ATG/TAA		+ 7
tRNA-Ser^AGN^ (S_1_)	7164–7225	62			GCT	+ 8
tRNA-Trp (W)	7234–7300	67			TCA	+ 13
*cox1*	7314–8942	1629	542	GTG/TAG		+ 10
tRNA-Thr (T)	8953–9015	63			TGT	0
*rrnL*	9016–9998	983				0
tRNA-Cys (C)	9999–10,065	67			GCA	0
*rrnS*	10,066–10,833	768				0
*cox2*	10,844–11,436	603	200	ATT/TAA		+ 13
*nad6*	11,450–11,905	456	151	ATG/TAG		+ 10
tRNA-Tyr (Y)	11,916–11,982	67			GTA	+ 2
tRNA-Leu^CUN^ (L_1_)	11,985–12,048	64			AAG	0
tRNA-Ser^UCN^ (S_2_)	12,049–12,117	69			TGA	+ 4
tRNA-Leu^UUR^ (L_2_)	12,122–12,186	65			TAA	+ 12
tRNA-Arg (R)	12,199–12,262	64			TCG	0
*nad5*	12,263–13,852	1590	529	GTG /TAG		+ 47
DNCR1	13,890–14,068	169				0
tRNA-Glu (E1)	14,069–14,130	62			TTC	0
DNCR2	14,131–14,299	169				0
tRNA-Glu (E2)	14,300–14,361	62			TTC	0
DNCR3	14,362–14,530	169				0
tRNA-Glu (E3)	14,531–14,592	62			TTC	0
DNCR4	14,593–14,761	169				0
tRNA-Glu (E4)	14,762–14,823	62			TTC	0
DNCR5	14,824–14,992	169				0
tRNA-Glu (E5)	14,993–15,054	62			TTC	0
DNCR6	15,055–15,223	169				0
tRNA-Glu (E6)	15,224–15,285	62			TTC	0
DNCR7	15,286–15,454	169				0
tRNA-Glu (E7)	15,455–15,516	62			TTC	0
LNCR	15,517–16,802	1286				0
tRNA-Gly (G)	16,803–16,869	67			TCC	+ 3

*SNCR* Small non-coding region, *DNCR* Duplicate non-coding region, *LNCR* Large non-coding region

^a^The inferred length of Amino Acid (aa) sequence of 12 protein-coding genes

^b^Ini/Ter codons: initiation and termination codons

^c^In: Intergenic nucleotides (between the current gene and the next gene)

**Table 2:** Nucleotide composition and skews of *Strigea falconis* mitochondrial genome

Gene	Nucleotide frequency				A + T (%)	AT-skew	GC-skew
	A (%) G (%) T (%) C (%)						
*atp6*	11.6	22.7	56.2	9.5	67.8	-0.658	0.410
*cox1*	16.3	24.0	47.6	12.1	63.9	-0.490	0.330
*cox2*	19.6	26.5	44.4	9.5	64.0	-0.388	0.472
*cox3*	14.3	25.2	51.7	8.8	66.0	-0.567	0.482
*cytb*	16.7	25.3	48.4	9.6	65.2	-0.496	0.451
*nad1*	15.6	26.0	50.7	7.7	66.2	-0.530	0.541
*nad2*	11.7	23.8	57.0	7.4	68.7	-0.659	0.524
*nad3*	15.4	24.9	53.8	5.9	69.2	-0.555	0.619
*nad4*	12.3	25.0	54.0	8.7	66.3	-0.629	0.484
*nad4L*	20.8	24.2	48.5	6.5	69.3	-0.400	0.577
*nad5*	11.8	24.9	54.3	9.0	66.1	-0.643	0.469
*nad6*	14.9	23.0	53.1	9.0	68.0	-0.562	0.438
*rrnS*	22.8	25.6	39.9	11.7	62.7	-0.273	0.373
*rrnL*	22.4	25.4	41.6	10.7	64.0	-0.300	0.408
tRNAs	22.2	26.8	40.1	10.9	62.3	-0.287	0.421
NCR	21.1	29.2	42.6	7.2	63.7	-0.338	0.606
Total	17.3	25.8	47.8	9.1	65.1	-0.469	0.478

### Protein-coding genes and codon usage

The 12 PCGs collectively span 9,348 bp and encode 3,116 amino acids. The initiation codons are ATG (*n* = 7) and GTG (*n* = 5). Canonical termination codons (TAA, TAG) are used by nine PCGs, whereas *cox3*, *cytb*, and *nad2* terminate with an incomplete ‘T’ codon, which is presumably converted to a full TAA stop codon via post-transcriptional polyadenylation (Table 1). Analysis of codon usage revealed a strong preference for A + T-rich codons. The most frequently used codons were TTT (Phe) and TTA (Leu), while codons with C/G in the third position were rarely used (Table 3).

**Table 3:** Relative Synonymous Codon Usage (RSCU) of four Strigeidae representatives

Amino acid	Codon	RSCU (%)				Amino acid	Codon	RSCU (%)			
		Sf	Ap	Com	Cam			Sf	Ap	Com	Cam
Phe	TTT	1.90	1.91	1.6	1.66	Tyr	TAT	1.85	1.83	1.5	1.67
Phe	TTC	0.10	0.09	0.4	0.34	Tyr	TAC	0.15	0.17	0.5	0.33
Leu	TTA	1.63	2.56	2.55	1.75	Stop	TAA	0.67	0.6	1.04	0.94
Leu	TTG	3.31	2.4	1.78	1.58	Stop	TAG	1.33	1.4	0.96	1.06
Leu	CTT	0.92	0.77	1.09	1.2	His	CAT	1.96	1.8	1.67	1.58
Leu	CTC	0.01	0.06	0.15	0.22	His	CAC	0.04	0.2	0.33	0.42
Leu	CTA	0.02	0.12	0.28	0.68	Gln	CAA	0.32	0.59	0.43	0.8
Leu	CTG	0.12	0.1	0.16	0.57	Gln	CAG	1.68	1.41	1.57	1.2
IIe	ATT	1.86	1.93	1.72	1.6	Asn	AAT	1.84	1.76	1.78	1.93
IIe	ATC	0.14	0.07	0.28	0.4	Asn	AAC	0.16	0.24	0.22	0.07
Met	ATA	0.69	0.9	0.93	1.12	Lys	AAA	0.60	0.74	0.94	1.03
Met	ATG	1.31	1.1	1.07	0.88	Lys	AAG	1.40	1.26	1.06	0.97
Val	GTT	2.54	2.46	2.48	1.94	Asp	GAT	1.90	1.84	1.85	1.89
Val	GTC	0.09	0.19	0.38	0.33	Asp	GAC	0.10	0.16	0.15	0.11
Val	GTA	0.34	0.56	0.51	0.67	Glu	GAA	0.24	0.35	1.14	0.49
Val	GTG	1.03	0.79	0.63	1.06	Glu	GAG	1.76	1.65	0.86	1.51
Ser	TCT	3.58	3.8	1.79	1.74	Cys	TGT	1.91	1.84	1.61	1.71
Ser	TCC	0.21	0.2	0.62	0.17	Cys	TGC	0.09	0.16	0.39	0.29
Ser	TCA	0.43	0.46	1	0.51	Trp	TGA	0.68	0.85	0.76	0.73
Ser	TCG	0.80	0.42	0.75	0.43	Trp	TGG	1.32	1.15	1.24	1.27
Pro	CCT	2.64	2.25	1.79	1.79	Arg	CGT	2.89	3.2	1.62	1.25
Pro	CCC	0.19	0.54	0.53	0.84	Arg	CGC	0	0.12	0.43	0.54
Pro	CCA	0.47	0.45	0.74	0.84	Arg	CGA	0.18	0.49	0.85	0.6
Pro	CCG	0.71	0.76	0.95	0.53	Arg	CGG	0.92	0.18	1.11	1.61
Thr	ACT	2.91	2.63	1.49	2	Ser	AGT	2.08	1.82	1.66	1.91
Thr	ACC	0.05	0.25	1.25	0.44	Ser	AGC	0.14	0.18	0.48	0.51
Thr	ACA	0.16	0.46	0.9	0.89	Ser	AGA	0.23	0.67	0.56	1.02
Thr	ACG	0.88	0.66	0.36	0.67	Ser	AGG	0.53	0.44	1.14	1.7
Ala	GCT	2.90	3.06	1.69	2.05	Gly	GGT	3.09	2.52	1.95	1.92
Ala	GCC	0.11	0.17	0.96	0.19	Gly	GGC	0.11	0.2	0.36	0.24
Ala	GCA	0.25	0.34	0.62	0.56	Gly	GGA	0.18	0.53	0.62	0.63
Ala	GCG	0.74	0.43	0.73	1.21	Gly	GGG	0.62	0.75	1.07	1.20

Excluding abbreviated stop codons (TA and T)

*Stop *Stop codon., *SfStrigea falconis*, *ApApharyngostrigea pipientis*, *ComCotylurus marcogliesei*, *Cam Cardiocephaloides medioconiger*

### Ribosomal and transfer RNA genes

**Fig. 2:**
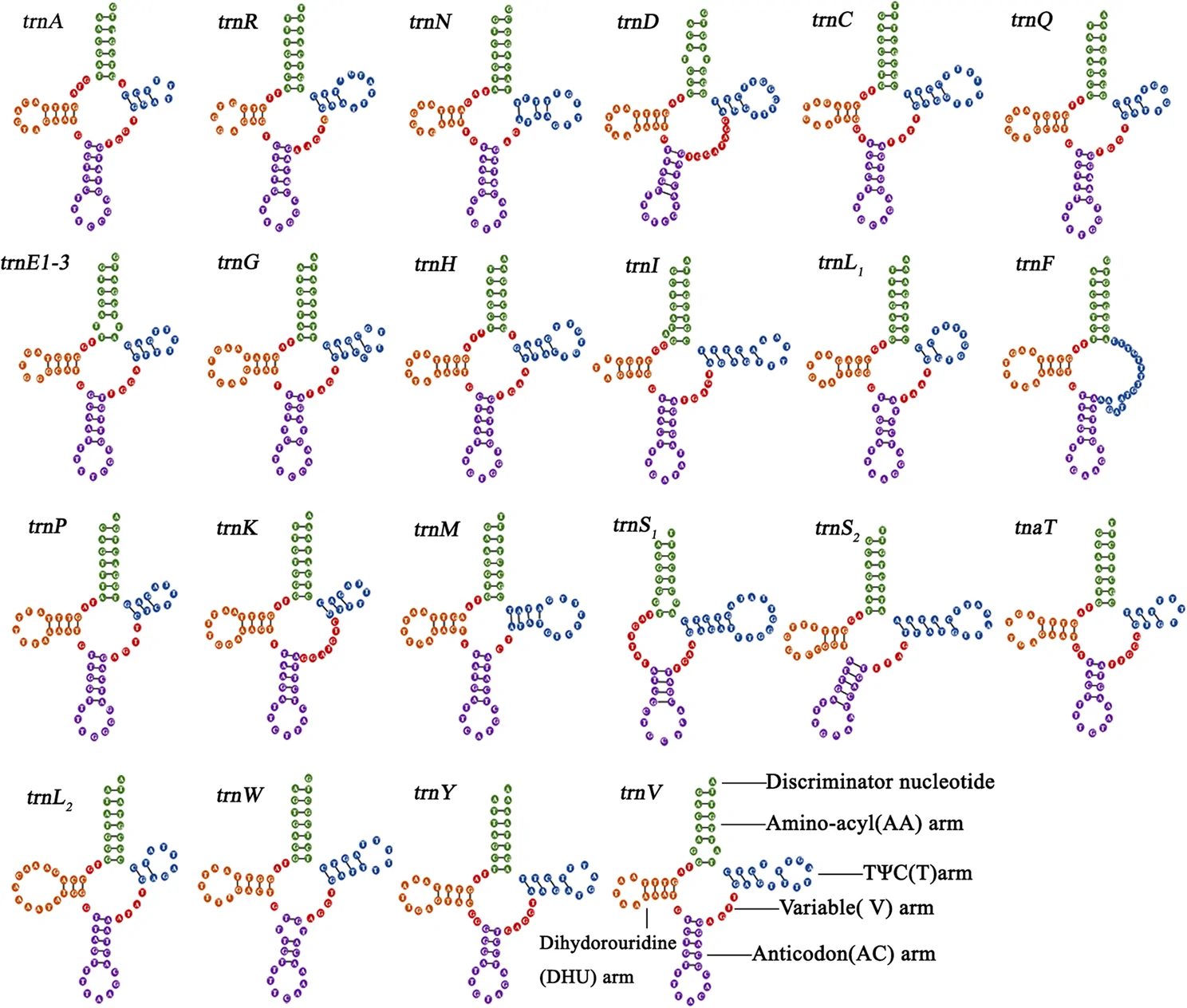
tRNA secondary structures in the mitochondrial genome of *Strigea falconis*

The *rrnL* (983 bp) and *rrnS* (768 bp) genes are located between *trnT* and *cox2*, separated only by *trnC*. The mt genome contains 22 typical tRNA genes, and six additional duplicated *trnE* genes. The tRNAs range from 62 to 69 bp. Most tRNAs could be folded into the canonical cloverleaf secondary structure, with two exceptions: *trnS*_*1*_(AGN) lacks the dihydrouridine (DHU) arm, and *trnP* lacks the TΨC arm (Fig. 2), features commonly observed in platyhelminth mt genomes [16, 17].

### Tandem repeat region containing duplicated *trnE* genes

**Fig. 3:**
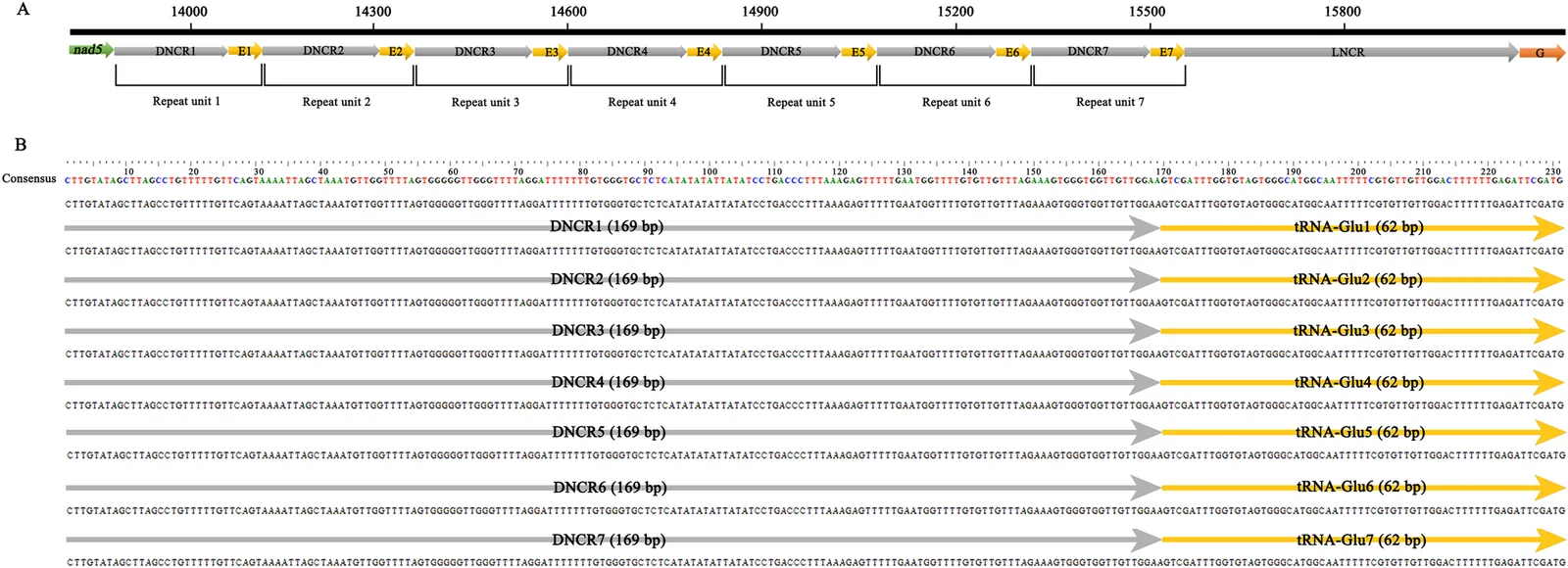
Diagram of insertions at the junction of non-coding region and tRNA-Glu. **A** The tandem repeat region found in *Strigea falconis*; (**B**) Sequence of the tandem repeat region in *S. falconis*, there are seven identical copies of the repeat unit

A striking feature of *S. falconis* mt genome is six tandemly repeated units, which is located between the *nad5* gene and the largest non-coding region (LNCR). Each repeat of 231 bp in length consists of a *trnE* gene of 62 bp followed by a 169 bp intergenic region (Fig. 3). The six repeats are 100% identical to each other. A χ^2^ test shows no statistically significant difference (χ² = 0.86, *p* = 0.35) in the usage of Glutamic Acid codons (GAA, GAG) between *S. falconis* and its close relative *A. pipientis* (which has a single *trnE*), suggesting the gene duplication has not yet driven a detectable shift in synonymous codon usage preference (Table 4).

**Table 4:** Chi-square test of codon usage between *Strigea falconis* with changed tRNA-Glu gene and *Apharyngostrigea pipientis*

Parameter	Glu		
	Species	GAA	GAE
Measured value	Sf	8	60
	Ap	23	57
Expected value	Sf	10	58
	Ap	10	59
Chi-square	0.870		
*P*	0.351		

*Sf Strigea falconis*, *Ap Apharyngostrigea pipientis*

### Phylogenetic relationships

**Fig. 4:**
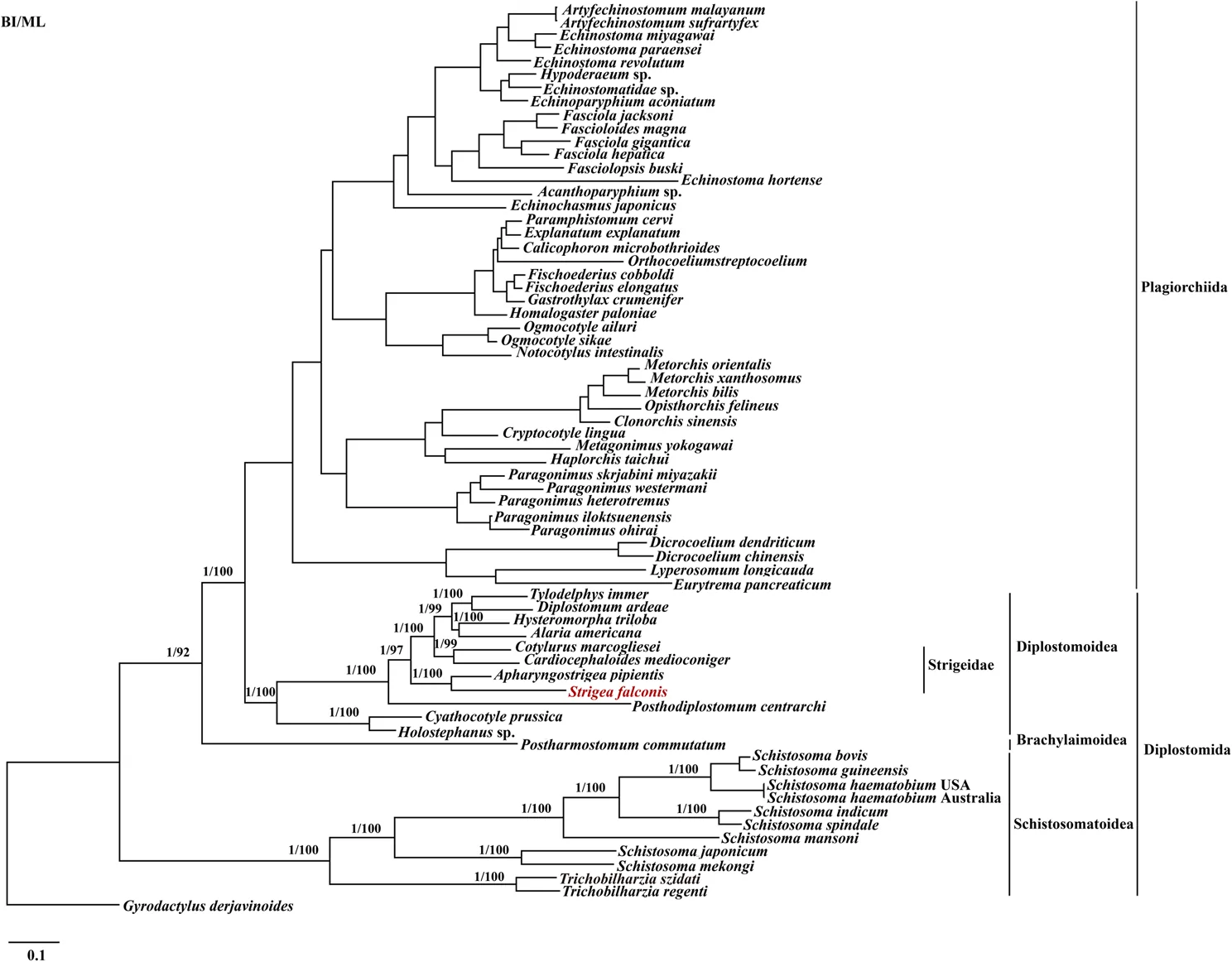
Phylogenetic relationships of Strigea falconis with other selected digeneans based on complete mitochondrial sequences. The concatenated amino acid sequences of 12 protein-coding genes were subjected to analysis by Bayesian analysis (BI) and Maximum likelihood (ML) using Gyrodactylus derjavinoides as an outgroup. The BI/ML order represents the arrangement of the main branch and posterior probability values

Both Bayesian analysis (BI) and Maximum likelihood (ML) analyses of the concatenated amino acid dataset derived from the 12 mt PCGs produced an identical topology with generally high nodal support (Fig. 4). Within the Diplostomida, three major clades corresponded to the superfamilies. The Schistosomatoidea formed a distinct, strongly supported monophyletic group (BPP = 1.0, BS = 100%). However, the remaining two superfamilies, Brachylaimoidea and Diplostomoidea, did not form a monophyletic group with Schistosomatoidea. Instead, they formed a strongly supported clade that was sister to the order Plagiorchiida (BPP = 1.0, BS = 100%).

Within Diplostomoidea, the newly sequenced *S. falconis* clustered with *A. pipientis*, forming a Strigeinae clade. However, the family Strigeidae was rendered paraphyletic by the placement of *C. medioconiger* and *C. marcogliesei*, which formed a sister group to the family Diplostomidae, rather than to other Strigeidae members. This topology received maximum support in both analyses (BPP = 1.0, BS = 100%), providing mt evidence for the non-monophyly of Strigeidae under the current taxon sampling.

## Discussion

The complete mt genome of *S. falconis* elucidated in the current study is the first one for the type genus *Strigea* in the family Strigeidae, which fills a critical gap in trematode systematics. Its gene content and arrangement are largely conserved with other sequenced Diplostomoidea, suggesting a stable ancestral architecture for this group [7, 10]. However, the mt genome also possesses a unique structural feature, specifically a tandemly repeated region containing a *trnE* gene and an intergenic region of 231 bp in length each repeat. Tandem repeats in the main non-coding region are a known source of size variation in metazoan mt genomes and are implicated in regulating replication and transcription [18, 19]. While tRNA gene duplications have been documented in other trematode, such as *Paragonimus westermani* [20], the repeat structure in *S. falconis*, with identical repeat units each containing a tRNA gene and an intergenic region, is highly unusual. This structure likely arose from a single duplication event of a *trnE*-containing segment, followed by subsequent tandem amplification, a process potentially mediated by slipped-strand mispairing during replication [21]. The 100% identity among the six repeats suggests this is a very recent evolutionary event or is maintained by gene conversion [22].

The finding that the duplication of *trnE* has not led to a significant shift in codon usage is intriguing. An increase in copy number of tRNA genes may enhance translational efficiency and drive codon usage bias [23], or may be subtle or time-lagged. Possible reasons for the latter include that: (1) insufficient time has passed for selection to act on synonymous sites in PCGs to favor codons corresponding to the duplicated tRNA; (2) the regulation of mt gene expression is complex, and other factors, such as translational machinery encoded by the nuclear genome, are more rate-limiting [24]. Future transcriptomic analyses could determine if all six *trnE* copies are expressed and processed, and broader sampling within the genus *Strigea* could reveal the evolutionary history of this repeat.

Our phylogenetic analyses, based on the mt amino acid dataset including the new *S. falconis* mt genome, provide additional mt evidence supporting the non-monophyly of Diplostomida under the present sampling. Schistosomatoidea, an early-diverging lineage, Brachylaimoidea and Diplostomoidea forming a sister group to Plagiorchiida, is consistent with several recent mt genomic studies [3, 5–7]. However, this stands in stark contrast to the traditional classification based on morphology and nuclear rDNA [2, 6, 9]. This deep incongruence between mt and nuclear phylogenies suggests a complex evolutionary history, potentially involving ancient rapid radiation, a process known to cause extensive incomplete lineage sorting (ILS) as a primary source of phylogenetic conflict, as demonstrated by large-scale mt genomic and nuclear datasets in vertebrates [25]. While such mito-nuclear discordance can arise from ancient rapid radiations, growing evidence from diverse vertebrates highlights ancient mt introgression and subsequent genome capture as a potent driver of such phylogenetic conflicts [26]. The consistent and highly supported mt genomic topology strongly suggests that the taxonomic rank and composition of Diplostomida require further reassessment.

Although the use of amino acid sequences can reduce the effect of synonymous substitution saturation, it does not fully eliminate potential biases caused by compositional heterogeneity, lineage-specific evolutionary rate variation, or model misspecification. In the present study, we did not perform formal compositional heterogeneity tests, amino-acid recoding analyses, or topology tests. Therefore, while the BI and ML analyses recovered a consistent and well-supported mitochondrial topology, the inferred relationships should be interpreted cautiously as mt evidence rather than as a definitive resolution of the species tree.

Our results also provide additional mt evidence for the non-monophyly of the family Strigeidae. The placement of *Cardiocephaloides* and *Cotylurus* in a sister clade to Diplostomidae, away from *Strigea* and *Apharyngostrigea*, is consistent with the previous work [7–9]. This suggests that the current family-level classification may not fully reflect the evolutionary relationships within Diplostomoidea. The morphological characters used to define these families (e.g., the structure of the tribocytic organ) may be homoplastic or have been misinterpreted [27]. Therefore, a systematic reassessment of Diplostomoidea integrating mt data, nuclear phylogenomics, and a re-evaluation of morphological traits is warranted.

Recently, Achatz and Tkach [28] proposed a revised classification of Diplostomoidea and suggested dissolving Strigeidae and incorporating its taxa within Diplostomidae. Our mt phylogeny, in which the traditional Strigeidae is not recovered as monophyletic, is broadly consistent with this reassessment. Nevertheless, we do not propose formal taxonomic changes here because our inference is based solely on mt genomes. Mitochondrial genomes represent a single, non-recombining, maternally inherited linkage group and may not always reflect the species tree. Future studies should include broader taxon sampling, independent nuclear phylogenomic datasets, and explicit sensitivity analyses, including assessments of compositional heterogeneity, recoding strategies, and topology tests, to evaluate the stability of these relationships.

## Conclusion

In conclusion, the first complete mt genome of *S. falconis* reveals a six-copy tandem repeat of *trnE*-containing units and provides an important new mt genomic resource for Diplostomoidea. Phylogenetic analyses based on mt protein-coding genes support the non-monophyly of Strigeidae and Diplostomida under the present mt dataset and taxon sampling. However, broader taxon sampling, independent nuclear phylogenomic data, and explicit tests of potential mt phylogenetic artefacts are needed to fully resolve these relationships and guide future taxonomic revision.

## Methods

### Trematode collection and species identification

The trematode specimens used in this study were collected from a *Circus cyaneus cyaneus* (Hen harrier) that had been rescued due to illness or injury and subsequently died. The bird was received and cared for by Beijing Wildlife Rescue and Rehabilitation Center, China. During necropsy, ten adult trematodes were recovered from the intestine. The trematode specimens were morphologically identified as *S. falconis* as previously described [29]. The specimens were then placed in the centrifuge tube and stored at − 80°C until use. Genomic DNA was extracted from individual flukes using the Wizard^®^ SV Genomic DNA Purification System (Promega, city, country) according to the manufacturer’s instructions. PCR targeting the mt *cox1* gene of the whole ten samples was performed using the primers (JB3: 5’-TTTTTTGGGCATCCTGAGGTTTAT-3’; JB4.5: 5’-TAAAGAAAGAACATAATGAAAATG-3’) as previously described [30], then followed by DNA sequencing.

### DNA sequencing, assembly, and verification

A DNA library was constructed by Novogene Bio-company (Tianjin, China) using 2 µg of DNA extracted from one adult worm. Paired-end sequencing (250 bp) was performed on the Illumina HiSeq 4000 platform. Raw sequencing data were processed to remove adapter sequences, highly duplicated reads, and reads containing ambiguous ‘N’ bases. The mt genome was assembled using Geneious Prime software [31]. One of the newly-obtained mt *cox1* gene sequence in the present study was regarded as a reference. The assembly parameters were set as follows: minimum overlap identity of 99%, minimum overlap length of 150 bp, and maximum gap size of 5 bp. To verify the accuracy of the mt genome assembly, long-range PCR was performed using five pairs of specific primers targeting conserved regions (Table S1). Among these fragments, the fifth long-range PCR amplicon, which spanned the tandem repeat region containing the duplicated *trnE* copies, was further subjected to Oxford Nanopore long-read sequencing. The filtered reads were mapped to the assembled using Geneious Prime software.

### Annotations

The mt genome of *S. falconis* was annotated using the MITOS web server [32]. Protein-coding genes (PCGs) and rRNA genes were identified by alignment with homologous sequences of a closely related species, *Apharyngostrigea pipientis*, using MAFFT 7.122 with the L-INS-I algorithm [33]. Potential secondary structures of the 22 tRNAs were predicted using ARWEN [34] and tRNAscan-SE [35]. The precise boundaries of PCGs were confirmed using ORF Finder (https://www.ncbi.nlm.nih.gov/orffinder/). Nucleotide sequences of the 12 PCGs were translated into amino acid sequences using MEGA v.12 [36]. AT/GC skews and relative synonymous codon usage (RSCU) were calculated [37]. A chi-square test was performed to compare the synonymous codon usage frequency of the *trnE* gene with that of *A. pipientis* to assess whether duplication events of the *trnE* gene influence codon usage preference.

### Phylogenetic analysis

In addition to the newly-described complete mt genome, 67 complete mt genomes (comprising amino acid sequences of 12 PCGs) from subclass Digenea were retrieved from GenBank for phylogenetic analysis (Table S2). The mt genome of *Gyrodactylus derjavinoides* (GenBank accession No. NC_010976), a monogenean fluke, was used as the outgroup. Amino acid sequences of the 12 PCGs were aligned using MAFFT [38] and concatenated into a single dataset. Conserved blocks within the alignment were selected using Gblocks 0.91b under default parameters [39].

The phylogenetic analyses were based on amino-acid sequences translated from the 12 mt PCGs. This strategy was used to reduce the potential effects of nucleotide substitution saturation, particularly synonymous substitutions at third codon positions, which can bias deep-level mt phylogenetic inference. Phylogenetic analyses were conducted using both BI methods and ML. BI was performed using MrBayes 3.2 [40], with eight Markov chains running for 1,000,000 generations in a Metropolis-coupled MCMC analysis, sampling trees every 100 generations. For ML analysis, bootstrap support values were generated with PhyML 3.0 using 100 rapid bootstrap replicates [41]. The best-fit substitution model (MtArt + I+G + F) was selected with ProtTest 2.4 under the Akaike Information Criterion [42]. The final phylogenetic tree was visualized using FigTree v1.4.4 (https://tree.bio.ed.ac.uk/software/figtree/).

No formal test of among-lineage compositional heterogeneity, posterior predictive model adequacy test, amino-acid recoding strategy, or topology test was performed in the present study. Therefore, although the use of amino acid sequences and the congruent topologies recovered by BI and ML provide support for the inferred mitochondrial relationships, possible effects of compositional heterogeneity, model misspecification, and other mitochondrial genome-specific biases cannot be fully excluded.

## Supplementary Information

Supplementary Material 1: Figure S1: PCR amplicons from the mitochondrial genome of Strigea falconis. Amplicons generated with the primers. M: DL5000 DNA marker, M0: Negative control, M1: Validation_01, M2: Validation_02, M3: Validation_03, M4: Validation_04, M5: Validation_05.

Supplementary Material 2: Table S1. Primers used for assembly validation. Table S2. Mitochondrial genome sequences of digeneans sequenced completely prior to the present study and used for phylogenetic analysis.

## Data Availability

The assembled mt genomes generated in the present study have been deposited in NCBI database under the following accession numbers: PP112874.
