# Supplementary material for: The first complete mitochondrial genome of Strigea falconis (Digenea: Strigeidae) reveals six tandemly repeated trnE-containing units and provides mt evidence for the non-monophyly of the family Strigeidae
Source: BMC Genomics. 2026 Jul 11;27:753. doi: 10.1186/s12864-026-13156-1 (PMC13576203; doi:10.1186/s12864-026-13156-1)
Supplement: Supplementary file 1 [file 12864_2026_13156_MOESM2_ESM.docx]

**Table S1** Primers used for assembly validation.

| **Primer no.** | **Direction** | **Sequence (5'-3')** | **Amplified region** | **Size (bp)** |
| --- | --- | --- | --- | --- |
| Validation_01 | F | TTTTGAGCGGCTACTGTATTGAC | Partial *cytb*-*nad4L-*partial *nad4* | ~ 1,770 |
|  | R | CCAACCCAGAACTATCATACAAA |  |  |
| Validation_02 | F | TCTTTGGGTCATGGCTTGTCTGC | Partial *nad4-*Q-SNCR-F-M-*atp6*-*nad2-*V-A-D-*nad1-*P-N-I-K-partial *nad3* | ~ 3,890 |
|  | R | ACGACTAAACTCCCGACTATCCA |  |  |
| Validation_03 | F | GGATAGTCGGGAGTTTAGTCGTG | Partial *nad3-*S_1-_W-*cox1-*T*-rrnL-*C*-*partial *rrnS* | ~ 3,740 |
|  | R | GAATCCTACTACCGAGTCCTGAA |  |  |
| Validation_04 | F | TTGTTTCAGGACTCGGTAGTAGGA | Partial *rrnS-cox2-nad6-*Y-L_1_*-*S_2_*-*L_2_-R-partial *nad5* | ~ 2,150 |
|  | R | TACGGGAGTCGGAGCACGCATAG |  |  |
| Validation_05 | F  R | AGAGTGCTTGTTTTCCATTTG  AGAAGTCAATACAGTAGCCGC | Partial *nad5*-DNCR1-E1-DNCR2-E2-DNCR3-E3-DNCR4-E4- NCR5-E5-DNCR6-E6-DNCR7-E7-LNCR-G-*cox3-*H-partial *cytb* | ~ 5,200 |
|  |  |  |  |  |

**Table S2** Mitochondrial genome sequences of digeneans sequenced completely prior to the present study and used for phylogenetic analysis.

| **Order** | **Family** | **Species** | **Size (bp)** | **GenBank accession no.** |
| --- | --- | --- | --- | --- |
| Diplostomida | Diplostomidae | *Tylodelphys immer* | 14193 | MH536513 |
|  |  | *Hysteromorpha triloba* | 13,855 | MH536511 |
|  |  | *Alaria americana* | 13,836 | MH536507 |
|  |  | *Diplostomum ardeae* | 14,037 | NC_049068 |
|  |  | *Posthodiplostomum centrarchi* | 14,561 | MH536512 |
|  | Cyathocotylidae | *Cyathocotyle prussica* | 13,665 | NC_039780 |
|  |  | *Holostephanus* sp. | 13,682 | OP082179 |
|  | Strigeidae | *Cardiocephaloides medioconiger* | 15,107 | MH536508 |
|  |  | *Cotylurus marcogliesei* | 13,815 | MH536509 |
|  |  | *Apharyngostrigea pipientis* | 14,348 | NC_059570 |
|  |  | ***Strigea falconis*** | **16,872** | **PP112874** |
|  | Brachylaimoidea | *Postharmostomum commutatum* | 13,799 | MH536512 |
|  | Schistosomatidae | *Trichobilharzia regenti* | 14,838 | DQ859919 |
|  |  | *Trichobilharzia szidati* | 14,293 | NC_036411 |
|  |  | *Schistosoma bovis* | 19,926 | CM014335 |
|  |  | *Schistosoma indicum* | 16,901 | NC_047240 |
|  |  | *Schistosoma spindale* | 16,901 | DQ157223 |
|  |  | *Schistosoma haematobium* USA  *Schistosoma haematobium* Australia | 15,003  33,418 | NC_0080742  MW067227 |
|  |  | *Schistosoma japonicum* | 14,085 | NC_002544 |
|  |  | *Schistosoma mekongi* | 14,072 | NC_002529 |
|  |  | *Schistosoma mansoni* | 14,415 | NC_002545 |
|  |  | *Schistosoma guineensis* | 15,267 | MK341584 |
| Plagiorchiida | Brachylaimoidea | *Postharmostomum commutatum* | 13,799 | NC_044643 |
|  | Dicrocoeliidae | *Lyperosomum longicauda* | 14,567 | NC_048467 |
|  |  | *Eurytrema pancreaticum* | 15,031 | KP241855 |
|  |  | *Dicrocoelium dendriticum* | 14,884 | NC_025280 |
|  |  | *Dicrocoelium chinensis* | 14,917 | NC_025279 |
|  | Echinochasmidae | *Echinochasmus japonicus* | 15,865 | NC_030518 |
|  | Echinostomatidae | *Artyfechinostomum malayanum* | 17,175 | NC_065766 |
|  |  | *Echinoparyphium aconiatum* | 14,865 | NC_067751 |
|  |  | *Echinostoma hortense* | 1,5048 | KR062182 |
|  |  | *Echinostoma revolutum* | 15,714 | NC_046395 |
|  |  | *Echinostoma miyagawai* | 14,416 | NC_039532 |
|  |  | *Artyfechinostomum sufrartyfex* | 14,567 | NC_037150 |
|  |  | *Echinostoma paraensei* | 20,298 | KT008005 |
|  |  | *Hypoderaeum* sp. | 14,180 | KM111525 |
|  |  | *Echinostomatidae* sp. | 13,985 | MN822299 |
|  | Fasciolidae | *Fasciola jacksoni* | 14,952 | KX787886 |
|  |  | *Fasciola gigantica* | 14,200 | OR123716 |
|  |  | *Fasciolopsis buski* | 14,833 | NC_030528 |
|  |  | *Fascioloides magna* | 14,047 | NC_029481 |
|  |  | *Fasciola hepatica* | 14,462 | NC_002546 |
|  | Gastrodiscidae | *Homalogaster paloniae* | 15,987 | NC_030530 |
|  | Gastrothylacidae | *Gastrothylax crumenifer* | 14,801 | NC_027833 |
|  |  | *Fischoederius cobboldi* | 14,256 | NC_030529 |
|  |  | *Fischoederius elongatus* | 14,120 | NC_028001 |
|  | Heterophyidae | *Haplorchis taichui* | 15,120 | MG972809 |
|  |  | *Cryptocotyle lingua* | 13,983 | NC_063968 |
|  |  | *Metagonimus yokogawai* | 15,258 | NC_023249 |
|  | Himasthlidae | *Acanthoparyphium* sp. | 14,191 | MG792058 |
|  | Notocotylidae | *Notocotylus intestinalis* | 14,317 | NC_059797 |
|  |  | *Ogmocotyle ailuri* | 14,642 | NC_071931 |
|  |  | *Ogmocotyle sikae* | 14,307 | NC_027112 |
|  | Opisthorchiidae | *Metorchis orientalis* | 13,834 | NC_028008 |
|  |  | *Metorchis xanthosomus* | 13,851 | NC_079699 |
|  |  | *Metorchis bilis* | 13,854 | NC_079698 |
|  |  | *Opisthorchis felineus* | 14,277 | NC_011127 |
|  |  | *Clonorchis sinensis* | 13,875 | NC_012147 |
|  | Paramphistomidae | *Paramphistomum cervi* | 14,014 | NC_023095 |
|  |  | *Orthocoelium streptocoelium* | 13,800 | NC_028071 |
|  |  | *Explanatum explanatum* | 13,968 | NC_027958 |
|  |  | *Calicophoron microbothrioides* | 14,028 | NC_027271 |
|  | Troglotrematidae | *Paragonimus westermani* | 20,273 | CM017921 |
|  |  | *Paragonimus skrjabini miyazakii* | 17,591 | NC_067029 |
|  |  | *Paragonimus iloktsuenensis* | 14,827 | NC_071218 |
|  |  | *Paragonimus heterotremus* | 13,927 | NC_039430 |
|  |  | *Paragonimus ohirai* | 14,818 | NC_032032 |
| Gyrodactylidea | Gyrodactylidae | *Gyrodactylus derjavinoides* | 14,741 | NC_010976 |

Note: The new mt genome sequenced and reported in the present study is bold.
